# Thoracic endovascular aortic repair with left subclavian artery coverage without prophylactic revascularisation—early and midterm results

**DOI:** 10.1007/s00423-014-1186-6

**Published:** 2014-04-26

**Authors:** J. Wojciechowski, L. Znaniecki, K. Bury, J. Rogowski

**Affiliations:** Department of Cardiac and Vascular Surgery, Medical University of Gdansk, ul. Debinki 7, 80-211 Gdansk, Poland

**Keywords:** TEVAR, Thoracic endovascular aortic repair, Aorta, Aneurysm, Dissection, Left subclavian artery

## Abstract

**Background:**

The management of the left subclavian artery when coverage is necessary during thoracic aorta endografting remains a matter of debate.

**Materials and methods:**

A retrospective analysis of a single-centre experience with thoracic endovascular aorta repair (TEVAR) was performed. Between April 2004 and October 2012, 125 cases of TEVAR were performed. The analysis focused on patients who required coverage of the left subclavian artery (LSA). We analysed mortality and morbidity with special attention to the rates of cerebrovascular accidents (CVAs) and spinal cord ischaemia (SCI) in the early and midterm.

**Results:**

Of the 125 patients, 53 (42 %, group A) required an intentional coverage of the LSA to obtain an adequate proximal seal for the endograft; the remaining patients constituted group B. None of the patients in group A had protective LSA revascularisation prior to TEVAR. The primary technical success rate was 79.2 vs. 90.3 % (group A vs. group B, *p* = 0.08), and the primary clinical success rate was 77.4 vs. 82 % (group A vs. group B, *p* = 0.53). The 30-day mortality rate was 11.3 vs. 11.1 % (group A vs. group B, *p* = 0.97). The 30-day morbidity was 7.5 vs. 13.9 % (group A vs. group B, *p* = 0.4). CVA occurred in 1.9 % of group A patients, compared to 1.4 % of patients from group B (*p* = 0.82). The SCI incidence rate was 0 vs. 1.4 % (*p* = 0.39). The mean follow-up of group A was 24.1 months (range 2–64.6 months, SD = 19). Additionally, the 1-year estimated survival was 85.5 %, and the 3-year estimated survival was 78 %. There were no midterm CVAs; one event of SCI occurred in the seventh post-operative month in group A.

**Conclusion:**

Our analysis, although retrospective and based on one institution experience, shows a realistic population of TEVAR patients. We prove that TEVAR with coverage of LSA origin can be accomplished with minimal neurological morbidity in this patient population. The study shows that LSA revascularisation is not mandatory before endograft deployment, especially in emergency settings. We also prove that although zone 2 TEVAR extends the proximal landing zone, it does not prevent type IA endoleaks from appearing. A multicentre randomised control trial with higher number of patients is necessary for proper, robust conclusion to be established.

## Introduction

Thoracic endovascular aortic repair (TEVAR) has rapidly become an accepted treatment option for numerous aortic pathologies [1–4]. An important requirement for successful endografting is confirming the health of the aorta in the landing zone where the graft is deployed. Left subclavian artery (LSA) coverage is necessary to achieve the proximal seal in up to 50 % of patients treated with TEVAR [5–9]. The management of LSA revascularisation in this cohort of patients remains a matter of debate. A 2009 consensus from the Society of Vascular Surgery described the quality of the existing evidence on the performance of subclavian revascularisation in patients undergoing TEVAR as low (2C) [10]. A 2011 literature review from the European Association for Cardio-Thoracic Surgery, which resulted in the formulation of recommendation of prophylactic LSA revascularisation in elective patients, was based on numerous and heterogeneous series with small samples of patients [11]. Studies in support of routine preoperative LSA revascularisation show that the coverage of the LSA during TEVAR is associated with an increased risk of stroke, paraplegia and arm ischaemia. Other studies have shown that coverage of the LSA without prophylactic revascularisation is not associated with increased morbidity, supporting those results promoting selective LSA revascularisation during TEVAR [7, 12–14]. In this study, we analysed the consequences of intentional LSA coverage during TEVAR with special attention to the development of neurologic complications in a real life, non-selected (“all comers”) population.

## Materials and methods

Between April 2004 and October of 2012, 125 TEVAR procedures were performed in our department. Of those, 42 % (*n* = 53) required stent-graft (S-G) deployment with intentional coverage of the origin of the left subclavian artery (Ishimaru zones 0–2 [15], group A), and this population constituted our studied group. The remaining patients had no coverage of the LSA (Ishimaru zones 3 and 4 [15], group B). We retrospectively reviewed a prospectively maintained database, medical records and imaging studies. Patients’ characteristics are listed in Table 1. The outcome measures evaluated included the rate of cerebrovascular accident (CVA), spinal cord ischaemia (SCI), and mortality and overall morbidity in early, as well as midterm observations. CVA was defined as any stroke or transient ischemic event, regardless of the extent of recovery. SCI was defined as any transient or permanent paralysis or paresis at any time after TEVAR.

**Table 1:** Patients’ demographics

Variable	Group A (%)	Group B (%)	*p* value
	*n* = 53	*n* = 72	
Age, median ± SD years	61 ± 15.2	72 ± 14.4	0.06
Male gender	44 (83 %)	52 (72.2 %)	0.16
CAD	6 (11.3 %)	13 (18.1 %)	0.30
Prior MI	1 (1.9 %)	8 (11.1 %)	0.10
Diabetes	5 (9.4 %)	9 (12.5 %)	0.59
Renal failure	0 (0 %)	6 (8.3 %)	0.32
Hypertension	39 (73.6 %)	45 (62.5 %)	0.19
Prior AAA open repair	5 (9.4 %)	7 (9.7 %)	0.95

*SD* standard deviation, *CAD* coronary artery disease, *MI* myocardial infarction, *AAA* abdominal aortic aneurysm

Preoperative planning was performed on Apple MacBook Pro systems with OsiriX DICOM Viewer version 3.9.4 (Pixmeo Sàrl, Bernex, Switzerland). The decision on the LSA coverage was based on preoperative contrast enhanced computed tomography (CTA) of the neck and thorax, evaluating the anatomy of the thoracic aorta, aortic arch and carotid and vertebral arteries. The anatomy of the circle of Willis was not routinely assessed. To further evaluate the cerebrovascular anatomy, doubtful elective patients in this subgroup underwent preoperative carotid and vertebral artery duplex Doppler ultrasonography. Three-dimensional MPR image reconstructions were performed to obtain centreline measurements of the aortic lumen. In patients who did not undergo supplemental preoperative imaging, the arch vessel anatomy was evaluated intraoperatively before S-G deployment. In elective cases, a proximal landing zone of ≥15-mm length was required. In emergency cases, however, a ≥10-mm proximal aortic “neck” was deemed suitable. In cases with shorter necks, the decision to cover the LSA was made. To classify the proximal landing zones, the aortic arch map with zones 0–4 by Ishimaru [15] was applied. In cases that required zone 0 TEVAR, patients would initially attain aortic debranching by the implantation of a bifurcated bypass from the ascending aorta to the brachiocephalic trunk and LCCA; later, during the same procedure, the S-G would be deployed in zone 0. Patients requiring zone 1 deployment underwent right-to-left carotid-carotid bypass procedures using an 8-mm Dacron graft.

We practice the strategy of conditional prophylactic revascularisation of the LSA in patients with patent LIMA-LAD bypass, dominant left vertebral or hypoplastic right vertebral artery, patent left arm dialysis fistula or axillo-femoral bypass graft. Additionally, intraoperative indications for LSA revascularisation include symptoms of acute left arm ischaemia.

From 2004 to 2009, our endovascular team consisted of a vascular surgeon and an interventional radiologist, and the procedures were performed in an interventional radiology suite. From 2009 to the present, a team of vascular and cardiac surgeons has been performing elective cases using a mobile C-arm (Siemens ARCADIS Avantic), while emergency cases are performed in an interventional radiology suite using an Siemens Artis zee C-arm (vascular surgeon and radiologist) unless aortic debranching is required (in those cases, the procedures are performed in the surgical theatre with the use of mobile C-arm).

In total, 63 % of procedures were performed under general anaesthesia, and 37 % were performed using loco-regional anaesthesia and sedation. Rapid pacing was used in one patient during S-G deployment in the critical landing zone.

Mean procedure time was 119 min ranging from 60 to 210 min.

No intraoperative neurological monitoring was conducted. Post-operative neurological assessments occurred every 2 h in the first eight post-operative hours and then every 4 h in uncomplicated cases (by either treating or on-call surgeon or anaesthesiologist). Our institution practices a policy of selective spinal drainage; this was reserved for complicated cases only. In cases of SCI, spinal drainage and augmentation of the mean arterial pressure would be utilised.

The stent grafts used are described in detail in Table 2.

**Table 2:** Stent grafts used

Stent graft	Group A (%)	Group B (%)
Zenith TX2 (Cook Inc., Bloomington, IN)	34 (64.1 %)	45 (62.5 %)
E-vita Thoracic (JOTEC GmBH, Hechingen, Germany)	11 (20.7 %)	19 (26.4 %)
Valiant (Medtronic, Minneapolis, MN)	4 (0.7 %)	5 (6.9 %)
Relay (Bolton Medical, Sunrise, FL)	4 (0.7 %)	3 (4.2 %)

Thoracic stent-graft delivery was approached through the common femoral artery in 118 patients, through the common iliac artery in 4 and through abdominal aorta in 3 patients. In these cases, an 8-mm vascular prosthesis was implanted end to side toward either the right common iliac artery or abdominal aorta and was then used as a technical conduit.

Patient follow-up included history, physical examination and CTA. Patients were typically seen at 1, 3, 6 and 12 months and annually thereafter. In specific conditions, follow-up was adjusted accordingly. During follow-up, the patients were specifically assessed for neurologic changes and evidence of arm ischaemia. CTA was used to assess the durability of the stent-graft repair.

Categorical variables were analysed using a nonparametric *χ* ^2^ test. In instances where the *χ* ^2^ test was unreliable due to a small sample size, the Fisher exact method was employed to test the association. Continuous variables were presented as medians, and they did not show a Gaussian distribution. Survival was analysed with the Kaplan-Meier and life-table analysis methods. All statistical analyses were performed using Statistica 7.1 software (StatSoft Inc., Tulsa, OK).

*Primary technical success* was defined as the successful introduction and deployment of the device in the absence of surgical conversion, death in ≤24 h and type I or III endoleaks, or graft obstruction [16].

*Primary clinical success* was defined as the successful deployment of the endovascular device at the intended location without death as a result of aneurysm-related treatment, type I or III endoleaks, graft infection, thrombosis, aneurysm expansion, aneurysm rupture or conversion to open repair [16].

## Results

### Indications

There were 76 elective (25 in group A and 51 in group B) and 49 emergency (28 in group A and 21 in group B) procedures. Among these, 110 were primary and 15 were secondary procedures. The indications for treatment are listed in Table 3. No patient who required zone 2 coverage had absolute indications for LSA revascularisation, and therefore, no preoperative revascularisation of the LSA was performed.

**Table 3:** Indications for treatment

Indication	Group A (*n* = 53)			Group B (*n* = 72)			*p* value
	Elective, no. (%)	Emergency, no. (%)	All, no. (%)	Elective, no. (%)	Emergency, no. (%)	All, no. (%)	
	25 (47.2 %)	28 (52.8 %)		51 (70.8 %)	21 (29.2 %)		0.007
TAA (mean diameter 75 mm, range 53–110 mm)	18 (34 %)			44 (61.1 %)			0.0027
		5 (9.4 %)			6 (3 rTAA) (8.3 %)		0.83
			23 (43.4 %)			53 (73.6 %)	0.0006
Complicated type B dissection		16 (30.2 %)	16 (30.2 %)		8 (11.1 %)	8 (11.1 %)	0.008
TAT		7 (13.2 %)	7 (13.2 %)		4 (5.6 %)	4 (5.6 %)	0.24
Pseudoaneurysm	6 (11.3 %)		6 (11.3 %)	5 (6.9 %)		5 (6.9 %)	0.39
Aortic ulcer	1 (1.9 %)		1 (1.9 %)	2 (2.8 %)		2 (2.8 %)	0.78

*TAA* thoracic aorta aneurysm, *TAT* thoracic aorta transection

### Proximal landing zone

There was one (1.9 %) deployment in zone 0. The proximal landing zone was zone 1 in 3 patients (5.8 %) and zone 2 in 48 patients (92.3 %). In the remaining cases, stent grafts were landed in zones either 3 or 4.

The primary technical success rate in group A was 79.2 % (*n* = 42). Assisted primary success was obtained in one patient (1.9 %) who required additional procedure during the same hospital stay, with proximal S-G deployment, due to a large type IA endoleak. Secondary success was obtained in two patients (3.8 %). The first of these was a female patient treated on an emergency basis for aortic transection due to vehicle trauma. She required additional subclavian-carotid transposition and forearm fasciotomy for acute left arm ischaemia, which developed after emergency TEVAR with LSA coverage (procedure performed in hypovolemic shock). Another patient was also treated on emergency basis (complicated acute type B aortic dissection). In this case, where zone 2 deployment was intended, post-deployment aortography revealed the coverage of LCCA origin (accidental zone 1 deployment). The patient was taken to the operating theatre, and RCCA to LCCA bypass was performed immediately. However, he developed stroke, and mild hemiplegia was present when the patient woke up after the procedure and on the day of discharge from the hospital. One patient had type III endoleak. Thus, the overall technical success rate in group A was 84.9 % (*n* = 45).

In group A, five patients had type IA endoleaks on completion angiography. In three cases, the endoleaks were minor, and decision on conservative treatment was made; these endoleaks had sealed upon follow-up observation and were absent on last follow-up CTA observations, taken on the 10th, 18th and 26th months after the procedure, respectively. In one case, a minor endoleak sealed upon follow-up and was absent on the last CTA control, performed on the 28th month post-procedure. Further follow-up contact with the patient was lost for 1.5 years, but then, he was admitted to our service in an emergency setting with rapidly enlarging, symptomatic thoracic aortic aneurysm (TAA) due to S-G migration and recurrence of type IA endoleak. He was taken to surgery right away, and debranching of the aortic arch by implantation of bifurcated prosthesis from ascending aorta to brachiocephalic trunk and LCCA was performed. During the deployment of the proximal extension S-G element in zone 0, the aneurysm ruptured to the left pleural cavity, and patient died intraoperatively. The last patient had a persistent IA endoleak on angio-CT 20 months after the procedure but refused another surgery. The aneurysm enlargement to 74 mm was observed, and the patient is still under ambulatory surveillance. The initial rate of type IA endoleak was 9.4 % (*n* = 5).

The primary technical success rate in group B was 90.3 % (*n* = 65).

Three type IA endoleaks were observed on completion angiography in group B. Conservative management was used in two cases; the first patient refused secondary procedure—at 2.5 months, significant aneurysm enlargement and rupture were observed with subsequent death of the patient. In the other case, the endoleak was minor and no aorta-related MAE was observed. The third patient had secondary procedure with LSA coverage after 14 months with good clinical result (no further endoleaks). The initial rate of type IA endoleaks was 4.2 % (*n* = 3) in group B.

There were also three type IB endoleaks (4.2 %, *n* = 3). In one case, type IB endoleak was observed in a patient treated for ruptured TAA, the patient died on post-operative day 1 in symptoms of hypovolemic shock. The remaining two patients had secondary procedures with distal S-G extensions; in both cases, the treatment was successful, with complete resolution of type IB endoleaks.

In one case, intraprocedural distal migration of S-G occurred, SMA origin was covered with the S-G and open extraction of S-G was performed; the patient died on the first post-operative day.

Details are listed in Table 4.

**Table 4:** Type IA endoleaks

Patient	Status of endoleak, last CTA control	Clinical outcome
Group A		
1	Seal, 26th month	Death on 27th month, urinary bladder carcinoma
2	Seal, 28th month	Migration, TAA rupture on 44th month. Another S-G deployment, intraoperative death
3	Sealed	Uneventful
4	Sealed	Uneventful
5	Another S-G placement during same hospital stay, seal	Uneventful
6	Persistant IA endoleak	Aneurysm growth to 74 mm, patient refuses another procedure
Group B		
1	No seal	TAA rupture on 3rd month, death
2	Seal, 48th month	Death on 54th month due to acute pancreatitis
3	Persistant type IA endoleak	Patient refuses surgery

*S-G* stent graft, *TAA* thoracic aorta aneurysm

### Number of grafts used

In group A, 26 patients required only one element, while in 13 patients, two stent-graft elements were used to cover the entire pathology. In 18 cases, the graft length was higher than 200 mm. Median was 205 mm (min 134 mm, max 332 mm, standard deviation ±55 mm).

In group B, 40 patients were treated with one stent-graft element; in 23 patients, two elements were used; and in two cases, 3 S-G elements were necessary to treat the entire aortic pathology. In 38 cases, the combined length of covered aorta was longer than 200 mm with a median of 213 mm (min 127 mm, max 353 mm, standard deviation ±73 mm).

Results based on stent-graft types are described in Table 5.

**Table 5:** Results based on stent-graft type

	Group A					Group B				
	Death	Type IA endoleak	Type IB endoleak	CVA	SSS	Death	Type IA endoleak	Type IB endoleak	CVA	SCI
Zenith TX2 (Cook Inc., Bloomington, IN)	5				1	5	2	3	1	
E-vita Thoracic (JOTEC GmBH, Hechingen, Germany)		2				3	1			1
Relay (Bolton Medical, Sunrise, FL)	1	2								
Valiant (Medtronic, Minneapolis, MN)		1								
Combined	6	5			1	8	3	3	1	1

*CVA* cerebrovascular accident, *SSS* subclavian steal syndrome, *SCI* spinal cord ischaemia

In other cases, a retrospective review of the patients’ notes would not allow us to establish the graft length or number of elements used.

### Type II endoleaks

There were no type II endoleaks originating from covered LSA in group A. There was two type II endoleaks in group B.

### Primary clinical success

The 30-day mortality rate was 11.3 % in group A and 11.1 % in group B. Therefore, the primary clinical success rate was 77.4 % (*n* = 41) in group A and 82 % (*n* = 59) in group B. A detailed description of early mortality may be found in Table 6.

**Table 6:** Detailed 30-day mortality

Patient	Post-op day	Indications	Cause of death
Group A (*n* = 6, 11.3 %)			
1	24	TAA	MOF after MI with circulatory arrest on post-op day 4
2	Intraoperative	Ruptured TAA	Hypovolemic shock
3	23	TAT	MOF
4	21	Complicated type B dissection	MOF
5	0	Ruptured TAA	Hypovolemic shock, MOF
6	17	TAT, multitrauma	PE
Group B (*n* = 8, 11.1 %)			
1	10	Complicated type B dissection	Retrograde type A dissection
2	4	TAA	MI
3	1	TAA	Access site injury
4	1	Ruptured TAA	Hypovolemic shock
5	3	Complicated type B dissection	MOF
6	1	TAA	Intraprocedural SMA origin occlusion, S-G extraction, MOF
7	10	TAA	MOF
8	6	Symptomatic TAA	MI

*TAA* thoracic aortic aneurysm, *MOF* multiorgan failure, *MI* myocardial infarction, *TAT* thoracic aorta transection, *PE* pulmonary embolism

The overall 30-day morbidity was 7.5 % (*n* = 4) in group A and 13.9 % (*n* = 10) in group B. No access site complications developed in group A, and there were five access related complications in group B.

The 30-day mortality and morbidity are listed in Table 7.

**Table 7:** Thirty-day mortality and morbidity

Variable	Group A (*n* = 53)	Group B (*n* = 72)
Mortality	6 (11.3 %)	8 (11.1 %)
MI	0	2 (2.8 %)
Reverse type A dissection	0	1 (1.4 %)
Bleeding from access site	0	1 (1.4 %)
Intraprocedural distal migration, SMA occlusion, laparotomy and S-G extraction	0	1 (1.4 %)
MOF	4 (7.5 %)	2 (2.8 %)
Hypovolemic shock	1 (1.9 %)	1 (1.4 %)
PE	1 (1.9 %)	0
Morbidity	4 (7.5 %)	10 (13.9 %)
Stroke	1 (1.9 %)	1 (1.4 %)
Paraplegia	0	1 (1.4 %)
SSS	1 (1.9 %)	0
Left arm ischemia	1 (1.9 %)	0
MI	1 (1.9 %)	3 (4.2 %)
Access site complications	0	5 (6.8 %)

*MI* myocardial infarction, *SMA* superior mesenteric artery, *MOF* multiorgan failure, *PE* pulmonary embolism, *SSS* subclavian steal syndrome

### Early neurologic complications

One stroke arose during the early observation period (stroke rate of 1.9 %) in group A and one stroke was observed in group B (1.4 %). No transient or permanent paraplegia had developed in group A in the 30-day observation, compared to one incident of paraparesis in group B (transient–complete resolution was observed on follow-up visit on the third month). In comparison with the group with LSA left uncovered, the experimental group showed no statistical significance in terms of CVA (1.9 vs. 1.4 %, *p* = 0.82) or SCI incidence rate (0 vs. 1.4 %, *p* = 0.39).

### Midterm clinical success

**Fig. 1:**
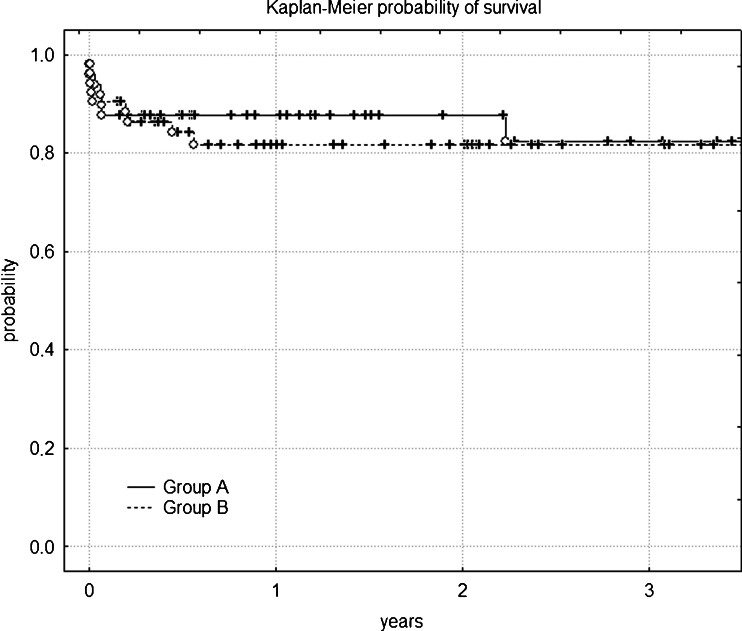
Kaplan-Meier survival outcomes for patients with and without coverage of LSA with TEVAR

The mean follow-up of group A was 24.1 months (range 2–64.6 months, SD = 19 months). The 1-year estimated survival was 87.7 % (standard error = 0.047) for group A vs. 81.7 % (standard error = 0.055) for group B (*p* = 0.64); the 3-year estimated survival was 82.3 % (standard error = 0.069) in the studied group and 81.7 % (standard error = 0.055) in group B (*p* = 0.65) (Fig. 1). The rate of late IA endoleaks in the studied group during the follow-up period was 9.4 %. Three type IA leaks (at 16, 20 and 43 months, respectively) were detected. One patient required zone 1 TEVAR after RCCA to LCCA bypass, and a follow-up CTA revealed sealed endoleak. The two other patients are on waiting lists for proximal extension procedures. Two secondary type IB leaks (6 and 18 months) were detected. Both were treated with secondary interventions with technical success. No type II leaks were found. No patients required open conversion.

In one patient, late paraplegia occurred (seventh month). He was treated for TAA with a history of prior AAA treatment with straight tube open repair. The MRI of his spine had revealed an ischemic focus on the T6–T8 level. In the follow-up CTA, a secondary IA endoleak was found in this patient (before paraplegia occurred).

Four cases of left arm ischaemia developed, and all were managed conservatively, with no need for revascularisation due to mild symptoms.

Four patients developed subclavian steal syndrome in midterm, with only one case becoming so profound that it required LSA to LCCA transposition. The effect of this procedure was good, with a complete resolution of symptoms and uneventful post-operative course.

## Discussion

In up to 50 % of cases of TEVAR, coverage of the LSA is necessary to obtain a seal and to prevent either type IA endoleaks or stent-graft migration [5–9]. In our population, the extension of landing zone to zones 0–2 by Ishimaru did not completely prevent type IA endoleakage, with a tendency to increased type IA endoleak rate (9.4 vs. 4.2 %, *p* = 0.41) in this group.

Chimney technique as well as scallop or multilayered prosthesis is an alternative for TEVAR with LSA coverage. These procedures require long-lasting technical preparation so they cannot be used in emergency cases. Another alternative may be the use of multilayered stents—however, nowadays, it delivers a lot of questions and has as many followers as opponents. The effectiveness of these method requires further investigation and trials; it cannot be recommended as a routine approach to this particular pathology [17].

Due to the extensive circulation provided by the LSA, coverage of the LSA can theoretically lead to grave complications, such as spinal cord ischaemia or cerebrovascular incidents, or to usually better tolerated chronic left arm ischaemia or subclavian steal syndrome. Although infrequent, acute upper extremity ischaemia has also been reported in the setting of LSA coverage [18]. However, such risks may be justified, especially in emergency situations in order to prevent mentioned endoleakage. In our population, there was a significant predominance of acute cases in group where LSA origin was covered (52.8 vs. 29.2 %, *p* = 0.007).

The management of intentional LSA coverage, particularly in elective setting, remains a matter of debate. Reports of a low incidence of left arm ischaemia shifted the pendulum towards the liberal coverage of the LSA as an attractive means of extending proximal landing zone in the early years of TEVAR [19, 20]. This complication, if it occurs, is well-tolerated in the vast majority of cases; if necessary, revascularisation may be implemented in an elective setting [21]. Still, reports of devastating neurological complications and studies showing an increased incidence of CVA and SCI have been published [22–25], calling for a reconsideration of the liberal LSA coverage policy.

However, the problem of additional risks associated with an extra procedure (LSA revascularisation) remains, and its necessity has been disputed [5, 26–28]. In fact, a high rate of complications, such as stroke (6.6 %) [26] and phrenic nerve injury (12.6 %) [24], has been reported. The most recent analysis published by Madenci et al. revealed a combined CVA and death rate of 5.3 % for isolated LSA reconstructions [29].

There was an attempt to standardise such care, and the Society for Vascular Surgery (SVS) [10] proposed recommendations based on selected trials and meta-analyses. Internally, the SVS recommendation graded the level of evidence as low—2C [10]. In a literature review from 2011, the European Association for Cardio-thoracic Surgery [11] strongly recommends prophylactic LSA revascularisation in elective patients. Still, level I evidence is non-existent.

The aim of this study was to examine the real-life results (tertiary centre providing aortic care to a population of ~2.3 million) of a selective LSA revascularisation strategy based on absolute indications only.

### CVA

In our group of patients with LSA coverage, one stroke occurred in a patient with an unintentional occlusion of left common carotid artery origin. It appears that this stroke (ischaemic focus in left frontal lobe) was directly related to left common carotid artery occlusion and not to LSA coverage.

However, a significant number of case series and meta-analyses have revealed an increased incidence of CVA in patients with LSA coverage without revascularisation [23, 30–32] and stroke protection by pre-TEVAR restoration of LSA flow [22, 33]. The reason for this is not clear, as many series did not report whether the strokes were in the posterior or anterior circulation. Posterior circulation strokes may indeed result from hypoperfusion caused by LSA coverage. However, anterior circulation strokes are instead often the result of embolisation caused by increased instrumentation in the aortic arch, which often is the case in zone 2 TEVAR procedures. Therefore, LSA coverage may be just an indirect marker of more advanced aortic disease with a higher hazard of embolisation. In fact, several recent papers explaining the reported stroke territory were found to support this theory [34–36]. The embolisation theory may also explain why numerous authors found LSA revascularisation to be ineffective in reducing the stroke rate [7, 12, 13, 23, 35, 37]. Furthermore, recent analysis by Maldonado et al. revealed that LSA revascularisation may even be harmful to certain patients (fourfold higher stroke incidence in females with LSA revascularisation) [14].

In our studied group, there were no other complications than the aforementioned stroke, meaning that neither embolisation strokes from the anterior circulation nor hypoperfusion strokes from the posterior circulation occurred.

Only 34 % (*n* = 18) of our patients were aged older than 70 years, making a relatively young population with a median age of 61. Secondly, only 43.4 % (*n* = 23) of patients had thoracic aortic aneurysm. Those two facts may indirectly indicate that our population had a low grade of either arch atheroma or thrombus and may explain the low incidence of CVA (an increased chance of CVA due to significant arch atheroma in patients >70 years of age was previously postulated [36, 38]).

### SCI

Our population of patients with LSA coverage without prior revascularisation had no SCI after a 30-day observation. This finding is in accordance with those of other studies in which selective LSA revascularisation was performed [7, 12, 13].

Apart from the debate on the influence of LSA coverage on the incidence of SCI, these are the well-known risk factors for paraplegia: coverage of a long segment of the aorta, use of more than three elements of stent grafts during the procedure, prior open abdominal aortic surgery and renal insufficiency [22].

Coverage of the aortic segment longer than 200 mm was found to be an independent risk factor for SCI by Kotelis [7]. The median length of the covered aorta in group A was 205 mm; however, the number of patients with a coverage of >200 mm may be more important. In our population, only 33.9 % (*n* = 18) of patients had segments longer than 200 mm covered, which may partially explain our good results. Additionally, none of our patients had more than two elements implanted; only the implantation of more than three elements significantly increases the risk of paraplegia, according to the EUROSTAR investigators [22].

We did not observe the influence of prior abdominal aortic repair on an increased early rate of SCI in the covered-LSA non-revascularised group. We had five patients with prior AAA surgery in the covered-LSA group, and none of them had SCI. However, all of these cases were elective. Czerny recently postulated that the coverage of two vascular territories in situations of hemodynamic instability may increase the chance of SCI [39]. The elective setting and no hemodynamic instability of these five cases may explain why no SCI occurred. Furthermore, hypogastric artery status would provide valuable additional insight into these cases; however, we have no such information in our case notes, hampering any conclusions.

Another factor playing a role in the explanation for the low SCI rate is that none of our patients of group A had renal insufficiency, which is known to be an independent risk factor [7, 22].

In the follow-up period, we recorded one case of paraplegia, which occurred in the seventh post-operative month. The paraplegia was due to spinal ischaemia with an ischemic focus at the level of T6–T8 and occurred in a patient who had an increased risk of early SCI based on the presence of one known risk factor (prior AAA open repair). In the follow-up CT, just before the occurrence of the paraplegia, a fresh type IA endoleak was found. We assume that, in this case, distal migration might have occurred (endoleak IA); the Adamkiewicz artery might have been occluded, resulting in paraplegia. The patient is ambulatory with crutches and is on a waiting list for proximal TEVAR extension.

Our results show that the implementation of a strategy of selective LSA revascularisation within a real-world, consecutive TEVAR population resulted in a low incidence of neurological complications. Low number of incidents did not allow to conduct a multivariate statistical analyses regarding SCI and CVA, which is a clear limitation of this study. Even if a relatively small sample size and heterogeneous pathology group may have biased our data, our per-procedure conclusions still stand.

However, we do foresee the necessity of further multicentre studies that report the combined morbidity of TEVAR with prophylactic LSA revascularisation vs. the morbidity of TEVAR without LSA revascularisation (preferably with all of the patients’ data included on an intention-to-treat basis) as the next step in solving the on-going debate.

## Conclusion

Our analysis, although retrospective and based on one institution experience, shows a realistic population of TEVAR patients. We prove that TEVAR with coverage of LSA origin can be accomplished with minimal neurological morbidity in this patient population. The study shows that LSA revascularisation is not mandatory before endograft deployment, especially in emergency settings. We also prove that although zone 2 TEVAR extends to the proximal landing zone, it does not prevent type IA endoleaks from appearing. A multicentre randomised control trial with higher number of patients is necessary for proper, robust conclusion to be established.
